# DynaNet: A Dynamic Feature Extraction and Multi-Path Attention Fusion Network for Change Detection

**DOI:** 10.3390/s25185832

**Published:** 2025-09-18

**Authors:** Xue Li, Dong Li, Jiandong Fang, Xueying Feng

**Affiliations:** 1 College of Information Engineering, Inner Mongolia University of Technology, Huhhot 010080, China; 13353741836@163.com (X.L.); 13354857638@163.com (X.F.); 2 Inner Mongolia Key Laboratory of Intelligent Perception and System Engineering, Hohhot 010080, China

**Keywords:** change detection, remote sensing images, contextual attention, multi-branch attention fusion, dynamic feature extractor

## Abstract

Existing change detection methods often struggle with both inadequate feature fusion and interference from background noise when processing bi-temporal remote sensing imagery. These challenges are particularly pronounced in building change detection, where capturing subtle spatial and semantic dependencies is critical. To address these issues, we propose DynaNet, a dynamic feature extraction and multi-path attention fusion network for change detection. Specifically, we design a Dynamic Feature Extractor (DFE) that leverages a cross-temporal gating mechanism to amplify relevant change signals while suppressing irrelevant variations, enabling high-quality feature alignment. A Contextual Attention Module (CAM) is then employed to incorporate global contextual information, further enhancing the discriminative capability of change regions. Additionally, a Multi-Branch Attention Fusion Module (MBAFM) is introduced to model inter-scale semantic relationships through self- and cross-attention mechanisms, thereby improving the detection of fine-grained structural changes. To facilitate robust evaluation, we present a new benchmark dataset, Inner-CD, comprising 800 pairs of 256 × 256 bi-temporal satellite images with 0.5–2 m spatial resolution. Unlike existing datasets, Inner-CD features abundant buildings in both temporal images, with changes manifested as subtle morphological variations. Extensive experiments demonstrate that DynaNet achieves state-of-the-art performance, obtaining F1-scores of 90.92% on Inner-CD, 92.38% on LEVIR-CD, and 94.35% on WHU-CD.

## 1. Introduction

Remote sensing change detection involves analyzing images taken at various times from the same locations to recognize alterations on the Earth’s surface [1]. This technology has a wide range of applications in multiple fields, including monitoring urban expansion [2], analysis of land-use change [3], agricultural resource surveys [4], protection of the ecological environment [5], and assessment of natural disasters [6]. With continuous advancements in remote sensing technology, change detection techniques have gradually expanded to include a variety of data sources, including high-resolution optical, SAR, and hyperspectral data. The availability of different data sources has facilitated the development and innovation of corresponding change detection methods [7,8,9,10,11]. In recent years, advancements in optical sensor technology have significantly increased the number of acquisitions of dual-temporal remote sensing image data, promoting in-depth research into automatic change detection methods and achieving a significant improvement in model performance [12,13].

**Figure 1:**
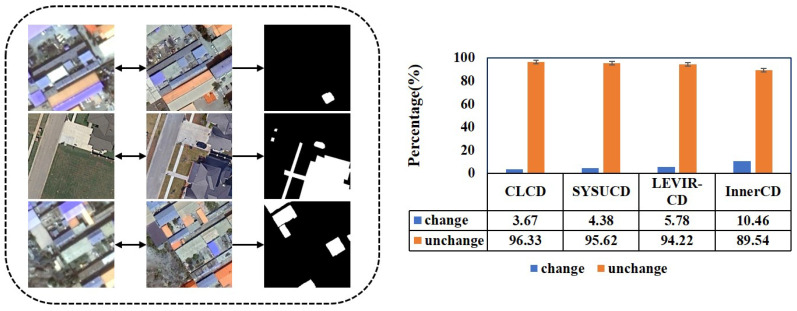
Partial samples of the Inner-CD dataset and the pixel imbalance distribution of other datasets. The left image presents a subset of the proposed Inner-CD dataset, while the right image illustrates the imbalanced distribution of changed and unchanged pixels across three datasets.

Change detection in remote sensing images can be considered a binary semantic segmentation task. In this process, each pixel is assigned a binary label to indicate whether the object of interest in that area has undergone any changes. In practical applications, various factors—including seasonal illumination changes, structural modifications of buildings, and differences in sensor and imaging conditions—often result in large non-change regions, presenting significant challenges for remote sensing change detection (RSCD). Additionally, the area of change regions is typically much smaller than that of non-change regions, requiring fine spatial details to improve detection accuracy. As shown in Figure 1, the left image presents a sample from our proposed Inner-CD dataset, while the right image illustrates the imbalanced distribution of change and non-change pixels in Inner-CD compared with three other datasets.

Conventional methods for change detection include Change Vector Analysis (CVA) [14] and Principal Component Analysis (PCA) [15]. CVA determines the type and extent of changes by analyzing the differences in pixel values and played an essential role in early studies. Conversely, PCA extracts the main components from images through linear transformation to highlight change areas. However, these methods have limitations, such as reliance on manually set thresholds, poor generalizability, and susceptibility to false positives or false negatives in complex environments. With advancements in machine learning techniques, sample-based approaches like Support Vector Machines (SVM) [16] and Random Forests (RF) [17] have been integrated into change detection. These methods can automatically learn change features, thus improving detection accuracy. However, SVM faces low computational efficiency when handling large-scale data and has limited classification ability for complex nonlinear change features. In contrast, RF can handle high-dimensional data but its generalization ability is still insufficient when dealing with complex land cover types and change patterns, limiting its adaptability to new datasets [18].

Recent advances in deep learning have driven substantial progress in remote sensing change detection. Among them, Convolutional Neural Networks (CNNs) have demonstrated remarkable capabilities in image processing, owing to their effective feature representation, and are widely adopted in this field [19,20,21,22,23,24,25,26,27]. CNNs automatically learn local features from images through convolutional layers, effectively capturing information such as texture and shape of land cover. For example, in land-use change detection, CNNs can accurately identify changes in different land covers, such as farmland, forests, and urban areas. Additionally, CNNs can process large-scale remote sensing data, demonstrating high computational efficiency and scalability.

However, CNNs also have limitations. Due to their local receptive field, CNNs mainly focus on regional features in images and cannot capture global contextual information. In some complex change scenarios, such as urban expansion or post-disaster change detection, relying solely on local features may not accurately assess the type and extent of changes. To address this issue, researchers are increasingly investigating the integration of Transformer architecture into remote sensing change detection [28,29,30,31,32,33,34]. The Transformer, through its self-attention mechanism, can simultaneously capture global and local information, providing excellent global modeling ability. For example, in flood disaster monitoring, the Transformer can precisely identify large submerged areas and detailed changes in buildings, roads, and other features within these regions.

Despite significant progress in existing remote sensing change detection (CD) methods, challenges persist in fine-grained change detection, as the effectiveness of deep learning models heavily relies on the quality of training datasets. Numerous prior studies have focused on creating high-quality datasets for change detection, such as the Land Cover Change Dataset (CLCD) [35], LEVIR-CD [23], and SYSU-CD [36]. However, most current change detection datasets are based on scenarios where one temporal image lacks buildings and the other contains dense buildings, with little focus on cases where both temporal images have many buildings that have undergone spatial or morphological changes. This poses a challenge to the adaptability of existing methods. Therefore, we propose a new Inner-CD dataset, specifically designed for research on building morphological and spatial structural change detection tasks, aiming to enhance the model’s ability to identify complex building change patterns.

To address these issues, this paper proposes DynaNet, a method combining dynamic feature change extraction with a multi-branch attention feature fusion network, and introduces a high-resolution building change detection dataset (Inner-CD). Specifically, DynaNet employs ResNet to extract multi-scale features from bi-temporal images, uses a Dynamic Feature Extractor (DFE) with cross-time gating to enhance change-related signals, applies a Contextual Attention Module (CAM) to leverage global contextual information, and integrates self- and cross-attention through a Multi-Branch Attention Fusion Module (MBAFM) to strengthen multi-scale feature relationships and improve building change detection. The main contributions are summarized as follows:

- We propose a Dynamic Feature Extractor (DFE) that enhances change-related features and suppresses background noise through a trans-temporal gating mechanism, enabling accurate extraction of fine-grained changes from bi-temporal images.
- A Contextual Attention Module (CAM) is designed to leverage global context, enhancing focus on key change regions while suppressing background noise, thereby improving the accuracy and robustness of fine change detection in complex environments.
- We introduce a Multi-Branch Attention Fusion Module (MBAFM) that models long-range dependencies and fuses multi-level features. By integrating self- and cross-attention, it enhances the structural relationships between buildings and their surroundings, improving change region recognition, boundary clarity, and robustness to noise.
- We provide a high-resolution Inner-CD dataset containing 600 pairs of 256 × 256 pixel images with a spatial resolution of 0.5–2 m to facilitate future research in building change detection.

## 2. Related Works

The rapid advancement of deep learning, particularly the extensive use of Convolutional Neural Networks (CNNs) in various domains, has greatly enhanced remote sensing image change detection technology. CNNs have become indispensable in change detection tasks due to their strong local feature extraction capabilities. Daudt et al. [19] proposed three basic models based on Fully Convolutional Networks (FCN) [37], which identify change regions through simple feature concatenation or differential calculation. Peng et al. [38] addressed error accumulation in feature difference computation by improving the UNet++ architecture and proposing an end-to-end change detection network with attention mechanisms and multiscale feature fusion, significantly improving detection performance. In addition, researchers have explored various optimization strategies for CNN structural improvements. For instance, Fang et al. [20] introduced a dense connection twin network based on UNet++ and implemented the Channel Attention Mechanism (CAM) to enhance the representation of fine-grained features. Zhang et al. [24] designed a deep supervision image fusion network that effectively combines spatial and guided attention mechanisms to improve feature representation. Han et al. [26] proposed a lightweight and efficient self-attention mechanism to integrate and optimize multi-scale feature representations. In change detection tasks, difference enhancement strategies are commonly employed to optimize network performance. For example, Zhang et al. [39] developed a differential pyramid to extract multi-scale difference feature maps. Lei et al. [40] developed a differential enhancement module that suppresses irrelevant noise and highlights actual change regions through CAM and residual connections. Chen and Shi [23] generated difference maps by calculating the Euclidean distance between features, effectively reducing false change phenomena and improving detection accuracy.

Meanwhile, lightweight networks have made significant progress in balancing computational efficiency with detection performance. Lei et al. [41] adopted multi-scale decoupled convolutions and a spatial–spectral feature cooperative strategy to significnatly enhance feature extraction efficiency and accuracy. Li et al. [31] improved feature aggregation and change recognition capabilities based on Mobile Networks, achieving excellent detection results while reducing computational costs. Liu et al. [42] proposed the EFICNN network, which uses an iterative VGG16 backbone with a Siamese structure combined with a 3D differential enhancement module, edge-guided attention module, and flow-guided fusion module, achieving excellent performance in change detection tasks.

Transformers, with their self-attention mechanism, have demonstrated significant advantages in modeling global dependencies and have gradually become the mainstream architecture in remote sensing change detection [43,44]. For example, Bandara et al. [29] proposed the ChangeFormer network, which significantly enhances multi-scale detail representation by combining hierarchical Transformer encoders and multilayer perceptron decoders. Zhang et al. [21] proposed the ChangeStar method, which uses object changes in unpaired images as supervisory signals, effectively improving model performance. Liu et al. [44] designed the Swin Transformer architecture, further strengthening global modeling abilities. Nevertheless, Transformers face challenges in local feature extraction and preserving fine details, particularly when processing high-resolution remote-sensing images. To address these shortcomings, researchers have attempted to combine CNNs and Transformers to create hybrid models. These models leverage the local feature extraction capabilities of CNNs while utilizing the global feature modeling advantages of Transformers, thus reducing computational complexity. For example, Chen et al. [45] proposed a hybrid model combining a ResNet encoder with a self-attention decoder, which achieved good results in change detection tasks. Transformer-based change detection networks have shown strong capabilities in modeling long-term dependencies. Liu et al. [35] proposed LGPNet, which extracts multi-scale building features through a local–global feature pyramid and further improves change detection performance by integrating attention mechanisms and transfer learning strategies. Chen et al. [33] proposed a method that transforms bi-temporal images into semantic tokens and models their relationships using a Transformer framework. Bandara and Patel [29] designed a Transformer-based change detection network that employs a twin Transformer encoder and multilayer perceptron decoder. Yan et al. [46] introduced a fully Transformer-based change detection network that enhances feature representation ability through global perception feature extraction and pyramid fusion. Zhang et al. [47] further designed a full Transformer network with a twin U-shape architecture to overcome CNN limitations in global change modeling.

Additionally, hybrid methods combining CNNs and Transformers have gained increasing attention. Xia et al. [48] proposed an edge-guided network that integrates ResNet with a Transformer encoder to optimize change feature extraction. Li et al. [49] combined Transformers with U-Net to extract global and local change information. Li et al. [31] proposed ConvTransNet, which integrates global and local features through a parallel CNN-Transformer structure and introduces a multi-scale framework to improve the detection of small-scale change regions. Notably, large foundational models such as Vision Transformer (ViT) [50] and CLIP [28] have achieved significant success in computer vision tasks and demonstrated strong generalization abilities in change detection tasks. Continued optimization of Transformer architectures and their integration with CNNs is expected to further expand their application potential in remote sensing change detection.

## 3. Method

### 3.1. Overall Architecture

**Figure 2:**
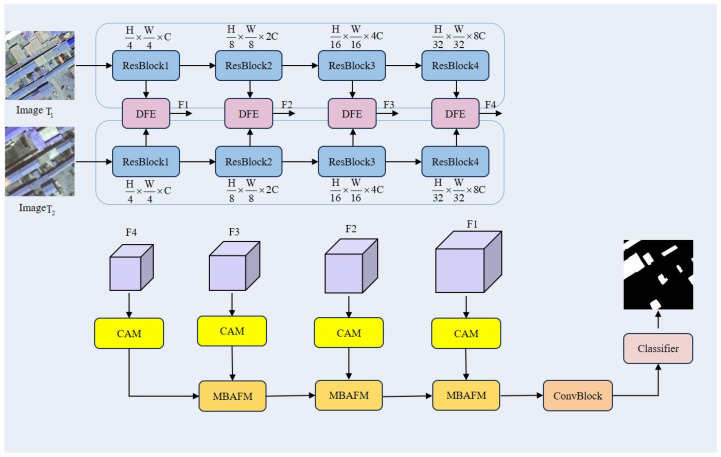
The overall architecture of DynaNet comprises four key components: the ResNet-18 backbone, the Dynamic Feature Extractor, the Contextual Attention Module, and the Multi-Branch Attention Fusion Module.

The proposed DynaNet adopts a typical encoder–decoder architecture consisting of three main components: the Dynamic Feature Extractor (DFE), the Contextual Attention Module (CAM), and the Multi-Branch Attention Fusion Module (MBAFM). The overall structure is illustrated in Figure 2. First, the bi-temporal remote sensing images T1 and T2 undergo feature extraction through ResNet-18—a shallower architecture within the Residual Network series, consisting of 18 layers with residual blocks and skip connections that mitigate gradient vanishing and decay issues. This step captures multi-scale spatial and semantic information from the bi-temporal images to form robust representations, thereby enhancing the perception of change regions. Next, these multi-scale features are input into DFE, where a differential operation generates a coarse change representation, which is then refined by deep DE convolution (DEConv) to capture finer spatial details. The extracted coarse change features are then processed by the CAM, which applies weighting based on global contextual information. This module enhances important regions while suppressing background noise, ensuring the model focuses on the most discriminative change areas. Subsequently, the bi-temporal features are further fused through the MBAFM, where the self-attention head captures intra-class relationships between different change regions, while the cross-attention head analyzes the interaction between change regions and the background. The self-attention head models the relationships between multiple change regions, while the cross-attention head focuses on the interaction between buildings and their surrounding environments, thereby improving the accuracy of building change detection. Finally, the fused features are optimized through a multilayer perceptron, which integrates global information to generate the final single-channel change prediction map.

### 3.2. Dynamic Feature Extractor

**Figure 3:**
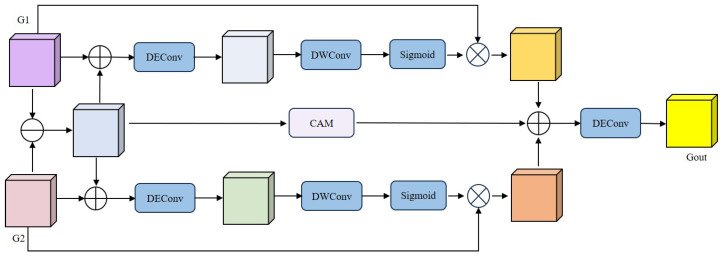
Structure of the proposed DFE.

Dual-temporal remote sensing imagery often contains complex ground objects and inconsistencies in imaging conditions (e.g., variations in weather, seasons, illumination changes, and temporary activities). These variations cause objects with the same semantics to exhibit different spectral features and generate multiple spectral variations. These factors give rise to a large number of meaningless variations, which increases the difficulty of dual-temporal feature fusion for change detection. Most existing methods rely on feature splicing or feature subtraction to reduce the interference of irrelevant changes. As a result, these methods perform poorly when dealing with different remote sensing change detection (RSCD) targets. To address this problem, we propose a DFE that employs a trans-temporal gating mechanism to guide the processing of change-specific features, selectively enhancing relevant changes while suppressing irrelevant variations. In this way, the DFE achieves high-quality dual-temporal feature fusion. As shown in Figure 3, the dual-temporal features $G_{1}$ and $G_{2}$ first undergo a differencing operation to obtain a rough change representation $G_{c}$.

(1) $$G_{c}=G_{1}-G_{2}$$

where − denotes elemental subtraction. $G_{c}$ is elementally spliced with $G_{1}$ and $G_{2}$, and then DEConv is performed to obtain $G_{c1}$ and $G_{c2}$ as follows:

(2) $$G_{c1}=DEConv\left(G_{c}⊕G_{1}\right)$$

(3) $$G_{c2}=DEConv\left(G_{c}⊕G_{2}\right)$$

Note that we chose Deep DE Convolution (DEConv) for two main reasons: (1) DEConv is able to significantly reduce the number of parameters of the model, thus reducing computational consumption; (2) DEConv captures finer spatial details by calculating the difference between the input feature maps and their feature maps processed with different convolution kernels. This allows the model to learn more details in the spatial dimension and thus generate more accurate weights. The weights are calculated as follows:

(4) $$W_{1}=Sigmoid\left(DWconv\left(G_{c1}\right)\right)$$

(5) $$W_{2}=Sigmoid\left(DWconv\left(G_{c2}\right)\right)$$

Finally, the previous rough change Fc is spliced through the Contextual Attention Module with the weighted features of the dual temporal phase to get our final output, where through the Contextual Attention Module, the model is able to weight the critical regions in the feature map to enhance focus on the change regions and suppress irrelevant information. It also ensures that the model can focus on areas that are critical for change detection, thus improving the detection accuracy.

(6) $$G_{out}=DEconv\left(\left(W_{1}⊗G_{1}\right)⊕\left(W_{2}⊗G_{2}\right)⊕\left(CAM\left(G_{c}\right)\right)\right)$$

where ⊗ denotes elemental multiplication.

### 3.3. Contextual Attention Module

In remote sensing change detection, due to complex ground objects and diverse imaging conditions, changes between two-time points are often subtle and noisy. Therefore, simple feature differentiation may lead to unnecessary interference. To address this issue, we propose a CAM that can identify the regions most relevant to change detection in the image and apply global contextual information to weight these regions. Through the global attention mechanism, the model not only focuses on the details of local regions but also integrates global information from the image, ensuring the capture of the most discriminative features. Specifically, CAM computes the weights for each position in the feature map, thus significantly improving change detection accuracy.

**Figure 4:**
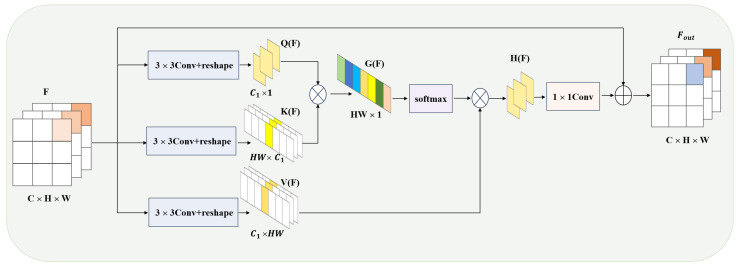
Structure of the proposed CAM.

Figure 4 illustrates the detailed structure of the Global Transformer Module. The input feature $F\in R^{C\times H\times W}$ first undergoes a 3 × 3 convolution and dimensional reconstruction to generate the query $Q\left(F\right)\in R^{C_{1}\times 1}$, key $K\left(F\right)\in R^{HW\times C_{1}}$, and value $V\left(F\right)\in R^{C_{1}\times HW}$, as shown in the following equation:

(7) VF,KF,QF∈Re(Conv3×3(F))

The process begins by combining $Q\left(F\right)$ and $K\left(F\right)$ and passing them through a softmax layer to compute the feature weights. These weights are then multiplied by the values to create the weighted feature map. In the final step, a 1 × 1 convolutional layer returns the features to their original dimensionality, and they are then combined with the original input features via concatenation to form the final result. The following illustrates this sequence of operations:

(8) $$G\left(F\right)=K{\left(F\right)}^{T}Q\left(F\right)\;$$

(9) H(F)=V(F)softmax(G(F))

(10) $$F_{out}=F+\alpha G\left(F\right)$$

where T denotes the transpose operation, α represents the 1 × 1 convolution, $G\left(F\right)\in R^{HW\times 1}$ denotes the fused features, $H\left(F\right)\in R^{C_{1}\times 1}$ represents the weighted features, and $F_{out}\in R^{C\times H\times W}$ is the output feature.

The Q in CAM acts as a channel query rather than a position query. The query vector is the feature selection from the key matrix channels. CAM can be roughly described as an attention mechanism that integrates channel and spatially weighted features with the input information.

### 3.4. Multi-Branch Attention Fusion Module

Compared to other parts of remote sensing image change detection, the regions of changed objects are typically small, making the restoration of spatial details of the change representation crucial [51]. During the feature fusion process, high-level change representations contain rich semantic information but have lower boundary quality, while low-level change representations capture spatial details but lack background information. Therefore, the common strategy adopted so far is to densely connect change representations at different scales to obtain both semantic information and spatial details. However, boundary and background information may introduce interference, leading to a decrease in change detection performance.

**Figure 5:**
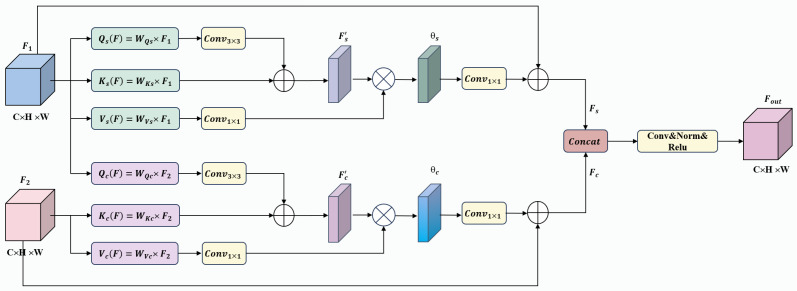
Structure of the proposed MBAFM.

For this reason, the MBAFM in this paper consists of self-attention headers and cross-attention headers, which are used to capture intra-class relationships between features at different levels and feature information highlighting the change region, respectively, as shown in Figure 5. Each header contains three trainable linear embedding layers (realized by 3 × 3 convolution and reshape operations), which are used as query, key and value generators, respectively, and the pair-by-pair formulas for query and key in the self-attention header and cross-attention header are as follows:

(11) $${F_{s}}^{1}\left(F_{1}\right)=Concat\left(Q_{s}\left(F_{1}\right),K_{s}\left(F_{1}\right)\right)$$

(12) $${F_{c}}^{1}\left(F_{1},F_{2}\right)=Concat\left(Q_{c}\left(F_{1}\right),K_{c}\left(F_{2}\right)\right)$$

where the subscripts s and c represent the self-attention and cross-attention heads, respectively. ${F_{s}}^{1}\left(F_{1}\right)$ and ${F_{c}}^{1}\left(F_{1},F_{2}\right)$ represent the initial self-attention features and cross features after pairwise computation of $F_{1}$ and $F_{2}$ using the query and key; $Q_{s}\left(\cdot \right)$ and $K_{s}\left(\cdot \right)$ are used to generate the queries and keys in the self-attention mechanism; $Q_{c}\left(\cdot \right)$ and $K_{c}\left(\cdot \right)$ are used to generate the queries and keys in the cross-attention mechanism. Next, the individual attention features of the two heads are computed as follows:

(13) $$\theta_{s}\left(F_{1}\right)=V_{s}\left(F_{1}\right)soft\max\left({F_{s}}^{1}\left(F_{1}\right)\right)$$

(14) $$\theta_{c}\left(F_{1},F_{2}\right)=V_{c}\left(F_{2}\right)soft\max\left({F_{c}}^{1}\left(F_{1},F_{2}\right)\right)$$

where the Softmax function is a commonly used activation function that transforms real-valued vectors into a probability distribution, where each element is between 0 and 1, and the sum of all elements equals 1. H is used to generate feature values in both the self-attention mechanism and the cross-attention mechanism. Residual learning is applied to each head, and the output is obtained as follows:

(15) $$F_{s}=W_{s}\theta_{s}\left(F_{1}\right)⊕F_{1}$$

(16) $$F_{c}=W_{c}\theta_{c}\left(F_{1},F_{2}\right)⊕F_{2}$$

where $W_{s}$ and $W_{c}$ are linear embeddings realized by 1 × 1 convolution, and the ⊕ operation is realized by residual concatenation through element-by-element addition.

The self-attention head captures long-range dependencies by calculating a weighted sum of features from all positions for a given position. This allows it to exchange information between multiple change regions, regardless of their distance, thereby modeling the intra-class relationships of these regions. At the same time, the self-attention mechanism helps to distinguish between different types of changes and further refine the boundaries of large-scale change areas, improving detection accuracy and enabling precise segmentation of building change areas. On the other hand, the cross-attention head extracts global structural information from land cover features, merging the interactions between buildings and their surrounding environments. Considering the close relationship between buildings and their environments, cross-attention helps to identify newly added or demolished buildings more accurately while reducing false detection caused by image noise or non-building structures (such as trees and road changes). In this paper, the features extracted by the self-attention head are fused with those extracted by the cross-attention head to form the final result:

(17) $$F_{out}=concat\left(F_{c},F_{s}\right)$$

### 3.5. Loss Functions

In this paper, we use the strategy of combining focal and Dice loss to address the problem of category inequality in change detection and enhance the accuracy of edge detection [52]. Together, these two loss functions complement each other, improving both robustness and detection accuracy. The combined loss function is shown below:

(18) Loss=FocalLoss+DiceLoss

Specifically, the formula for loss of focus is as follows:

(19) $$FocalLoss=-\alpha {\left(1-p_{t}\right)}^{\gamma }\log\left(p_{t}\right)$$

(20) $$p_{t}=\left\{\begin{array}{cc} p, & \mathrm{if}\;y=1\\ (1-p), & \mathrm{otherwise} \end{array}\right.$$

where, α and γ are two hyperparameters, α governs the weighting of positive and negative samples, while adjusts the model’s emphasis on difficult-to-classify samples (empirically set to α = 0.2 and γ = 3). The term denotes the predicted probability, and is the ground-truth binary label, with 0 signifying unchanged pixels and 1 indicating changed pixels. Furthermore, the expression for the Dice loss is as follows:

(21) $$D\mathrm{ice}Loss=1-\frac{2\cdot \left|P⋂G\right|+\mathrm{smoo}\mathrm{thing}}{\left|P\right|+\left|G\right|+\mathrm{smoo}thing}$$

where P represents the region predicted by the model and G represents the ground truth region. |P| and |G| are their respective areas (or pixel counts). The smoothing term is a small positive number used to ensure that the denominator is not zero when both P and G are empty regions, thus avoiding computational errors. Adding the smoothing term in cases of small areas or imbalanced classes helps improve numerical stability and prevents large fluctuations in the loss value.

## 4. Experimental Results and Analysis

### 4.1. Dataset Introduction

**Figure 6:**
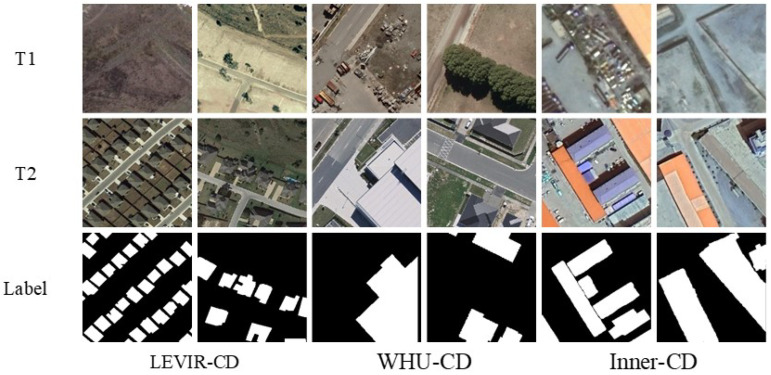
Selected samples from the three datasets.

The efficacy of the proposed DynaNet was validated through experiments on two widely used public building change detection (BCD) datasets, LEVIR-CD [23] (LEarning, VIsion and Remote sensing laboratory, Beijing, China) and WHU-CD [53] (Wuhan University Sigma Laboratory, Wuhan, China), alongside our proprietary Inner-CD dataset. For consistency with our small-batch learning strategy, all datasets were uniformly segmented into non-overlapping image patches of 256 × 256 pixels and subsequently divided at random into training, validation, and testing subsets. Figure 6 presents visual examples of bi-temporal input images and their corresponding change labels from each dataset.

(1) Inner-CD (Ours): Inner-CD is a newly curated high-resolution urban change detection dataset, meticulously developed to address fundamental limitations in existing datasets such as LEVIR-CD and WHU-CD. These traditional benchmarks typically simplify the change detection task to binary presence or absence scenarios, failing to adequately represent finer-grained structural evolutions and nuanced morphological alterations commonly encountered in real-world urban settings.

To bridge this critical gap and foster the advancement of structure-aware and content-sensitive change detection methodologies, Inner-CD features 600 meticulously curated bi-temporal image pairs, each with a resolution of 256×256 pixels. These images were extracted from multi-temporal Google Maps satellite imagery covering diverse urban and suburban landscapes in Hohhot and Ulanqab, Inner Mongolia, China. The imagery captures temporal spans ranging from 2 to 5 years, providing an authentic representation of urban morphological dynamics. The spatial resolutions within Inner-CD vary between 0.5 and 2 m, reflecting realistic variations in data quality and geographical conditions.

**Figure 7:**
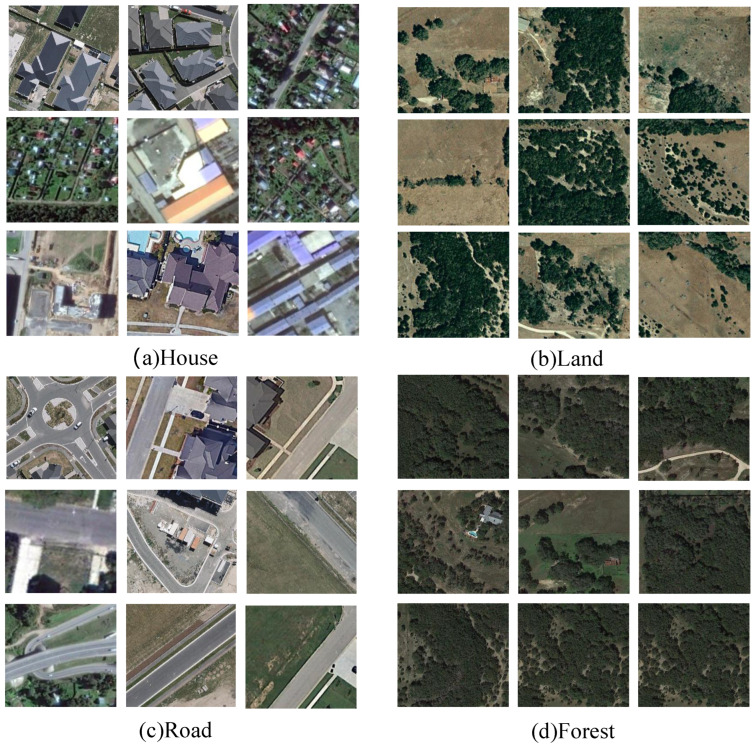
Illustrative examples from the Inner-CD dataset, depicting representative bi-temporal image pairs across the four primary change categories: House, Land, Road, and Forest. Subtle yet critical morphological variations are highlighted, including partial demolitions, expansions, land-use modifications, road alterations, and minor structural adjustments in forested regions. These examples underscore the dataset’s emphasis on fine-grained structural evolution and nuanced urban dynamics.

Distinctively, Inner-CD is characterized by high temporal symmetry, where the primary categories—House, Land, Road, and Forest—remain present across both temporal captures. Unlike conventional datasets that focus on simple appearance or disappearance events, Inner-CD emphasizes subtle intra-object variations, such as partial demolitions and incremental expansions in houses, nuanced alterations in land utilization, road modifications, and slight morphological changes in forested areas. Such fine-grained changes challenge models to move beyond straightforward detection tasks and engage in intricate structural comparisons, detailed spatial analysis, and semantic interpretation (as illustrated in Figure 7).

Each image pair within Inner-CD underwent rigorous annotation conducted by expert annotators using advanced GIS tools. Annotators precisely delineated change areas at a pixel-level, categorizing them into one of the four specified classes: House, Land, Road, and Forest, ensuring high-quality, accurate, and consistent annotations. To guarantee annotation reliability and minimize errors, a comprehensive multi-step validation approach was employed, incorporating cross-verification by multiple annotators and periodic oversight by senior quality assurance experts.

By significantly elevating the semantic complexity and spatial nuance, Inner-CD compels change detection models toward more sophisticated and robust capabilities, including nuanced morphological reasoning and intricate feature correlation analyses. This rigorous approach facilitates the development and benchmarking of advanced algorithms designed to operate reliably in realistic and morphologically complex scenarios.

Ultimately, Inner-CD serves not merely as another dataset but as a foundational resource intended to propel the field of urban change detection toward achieving higher standards of accuracy and robustness in recognizing intricate urban dynamics. It provides an ideal benchmark to rigorously assess models like DynaNet, particularly in their ability to effectively handle delicate structural changes, nuanced spatial transformations, and sophisticated inter-temporal correlations essential for practical real-world deployment.

(2) LEVIR-CD [23]: The LEVIR-CD dataset contains 637 high-resolution (1024 × 1024) image pairs with a 0.5-m spatial resolution, collected across multiple cities in Texas, USA. The temporal gap ranges from 5 to 14 years. Building types include villas, apartments, garages, and warehouses, but change patterns are typically large-scale insertions of buildings into previously empty regions, making detection tasks more binary and localized.

(3) WHU-CD [53]: This dataset consists of two large aerial images with dimensions of 32,507 × 15,354 pixels and a 0.3-m resolution. Similar to LEVIR-CD, it focuses on significant structural additions or removals, with relatively limited intra-object semantic variation. As such, although it supports general change detection, it lacks the temporal symmetry and spatial nuance present in Inner-CD.

### 4.2. Implementation Details and Evaluation Metrics

All experiments were conducted on a 64-bit Ubuntu 20.04 system equipped with an Intel Xeon(R) Gold 6430 CPU @ 16 GHz, an NVIDIA RTX 4090 GPU, and a deep learning environment configured with Python 3.10, PyTorch 2.2.0, and CUDA 11.8. (All equipment and environment configurations are managed on a computing power leasing platform developed by Shituo Cloud China (Nanjing) Technology Co., Ltd., Nanjing, China) We adopted Stochastic Gradient Descent (SGD) as the optimizer, owing to its stability and reliable convergence on large-scale datasets. A momentum of 0.9 was employed to accelerate convergence and smooth gradient updates. The initial learning rate was set to 0.05 with a weight decay of 0.00005, ensuring global search capability while mitigating overfitting. To simplify training and maintain stability, a fixed single-stage learning rate schedule was applied. The batch size was set to 8, considering the high resolution of remote sensing images and the resulting large GPU memory consumption. To compensate for the relatively small batch size, the training process was extended to 200 epochs. Overall, these parameter choices were made to balance convergence speed, model complexity, and generalization ability under the constraints of remote sensing change detection tasks and available hardware resources.

To assess the performance of the proposed algorithm, we employed a suite of standard metrics for change detection, namely precision (Pr), recall (Re), F1-score, and intersection over union (IOU). High precision helps reduce false positives, while high recall helps minimize false negatives. These metrics are composite indicators, with higher values indicating better performance. The formulas for calculating these metrics are as follows:

(22) $$F1=2\times \frac{Pr\times Re}{Pr+Re}$$

(23) $$Pr=\frac{TP}{TP+FP}$$

(24) $$Re=\frac{TP}{TP+FN}$$

(25) $$IOU=\frac{TP}{TP+FN+FP}$$

where TP and TN denote the number of pixels correctly classified as changed and unchanged, respectively. Conversely, FN refers to the count of pixels that are actually changed but were incorrectly predicted as unchanged, while FP is the count of pixels that are actually unchanged but were incorrectly predicted as changed.

### 4.3. Comparison Experiments

Comparison Methods: To benchmark the performance of DynaNet on high-resolution remote sensing change detection tasks, we conducted a comparative analysis against 13 established methods, which were grouped into three distinct classes. The initial group comprises models based on Convolutional Neural Networks (CNNs), such as FC-Siam-Diff [54], FC-Siam-Conc [19], SNUNet [20], StarCD-Net [54], and DTCDSCN [44]. The FC-Siam-Diff and FC-Siam-Conc models both utilize CNN architectures; however, FC-Siam-Conc further integrates Siamese networks and feature fusion strategies to improve its change detection capabilities. SNUNet, also a CNN-based method, adopts the U-Net architecture, effectively extracting local features, thereby improving detection accuracy. StarCD-Net is a CNN-based method that leverages StarNet and differential operators to enhance edge-aware change detection. The second category consists of Transformer-based methods, including DMATNet [55], BIT [33], Changeformer [29], and STANet [23]. BIT uses a Transformer architecture to capture global dependency information; Changeformer is designed explicitly for change detection in remote sensing images; and STANet improves model performance by introducing a temporal modeling mechanism. The third category includes hybrid frameworks combining CNN and Transformer, such as ConvTransNet [31], WNet [32] and LGPNet [35], which adopt strategies combining CNNs and Transformers. RDSF-Net [56] integrates CNN and Mamba—a state-space-model-based efficient global modeling structure—and employs residual wavelet transform for downsampling.

Quantitative Analysis: Table 1, Table 2 and Table 3 present the experimental results of DynaNet and other state-of-the-art methods on three datasets.

For the LEVIR-CD dataset, DynaNet outperforms all other evaluation metrics, achieving the highest F1-score (92.38 %), precision (93.45%), recall (91.46%), and IOU (84.57%). Compared to the second-ranked RDSF-Net, DynaNet achieves an F1-score that is 1.11% higher, precision that is 2.29% higher, recall that is 1.01% higher, and IOU that is 0.78% higher. This demonstrates that DynaNet offers a clear performance advantage on the LEVIR-CD dataset, detecting changes more accurately while maintaining high precision and recall.

For the WHU-CD dataset, DynaNet performs the best across three evaluation metrics, although it slightly trails Changeformer in precision. Specifically, DynaNet leads in F1-score (94.35%), recall (92.46%), and IOU (87.57%), while Changeformer outperforms in precision. However, its lower recall results in an overall performance that is still inferior to DynaNet. Compared to ConvTransNet, DynaNet leads in precision, recall, F1-score, IOU, and OA (Overall Accuracy) by 1.22%, 1.13%, 1.18%, 2.04%, and 0.07%, respectively, further confirming the superiority of DynaNet on the WHU-CD dataset.

For the Inner-CD dataset, DynaNet also performs strongly, achieving an F1-score of 90.92%, precision of 93.80%, recall of 87.84%, and IOU of 80.37%. DynaNet excels in F1-score, precision, recall, and IOU compared to existing methods. These results demonstrate that the proposed framework has excellent generalization and adaptability when handling building change detection tasks. This is primarily due to the general knowledge learned from two pre-trained models, allowing the model to effectively handle complex scenarios where buildings are abundant in both temporal images, and some undergo spatial position or morphological changes. This capability is particularly important for Inner-CD, where the change patterns are more complex and diverse than those in conventional datasets.

**Figure 8:**
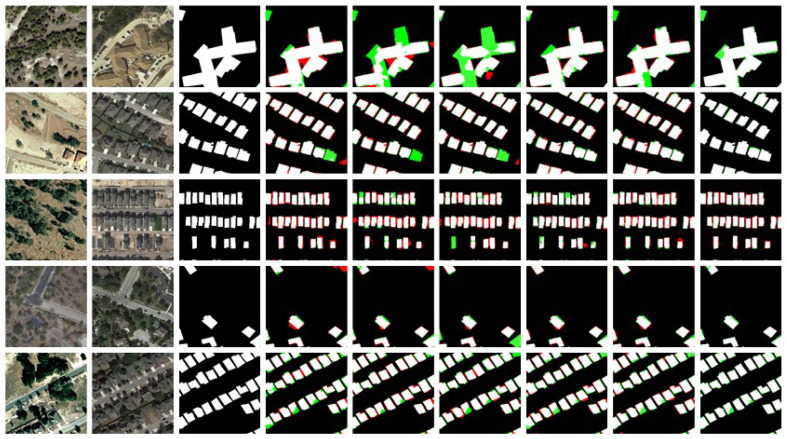
The LEVIR-CD dataset displays example outputs of DynaNet and other comparison methods. Pixels are visualized with color encoding, where white represents true positives (TP), black represents true negatives (TN), red represents false positives (FP), and green represents false negatives (FN).

**Figure 9:**
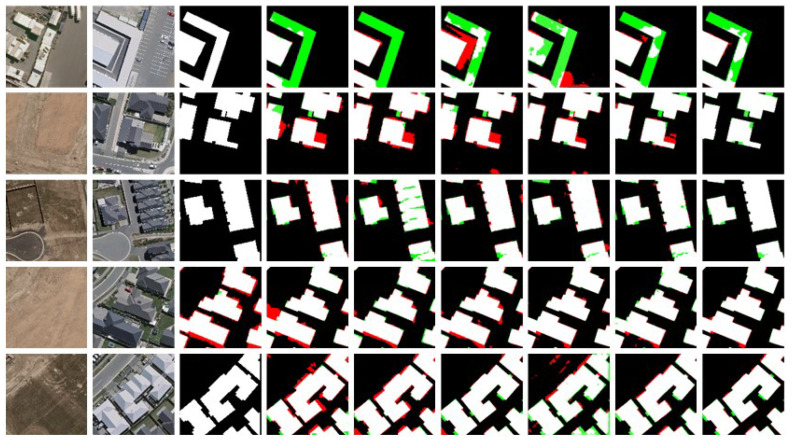
The WHU-CD dataset displays example outputs of DynaNet and other comparison methods. Pixels are visualized with color encoding, where white represents true positives (TP), black represents true negatives (TN), red represents false positives (FP), and green represents false negatives (FN).

**Figure 10:**
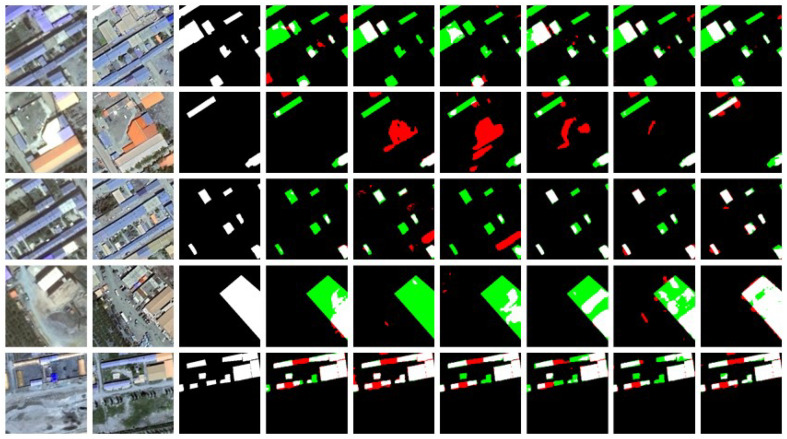
The Inner-CD dataset displays example outputs of DynaNet and other comparison methods. Pixels are visualized with color encoding, where white represents true positives (TP), black represents true negatives (TN), red represents false positives (FP), and green represents false negatives (FN).

Visualization Analysis: In the visualization analysis, black represents TN (True Negative), white represents TP (True Positive), green represents FN (False Negative), and red represents FP (False Positive). Figure 8 shows the visualization results of change detection using different methods on the LEVIR-CD dataset. Figure 9 and Figure 10 display similar results on the WHU-CD and Inner-CD datasets. In the visualization images, the sequence from left to right shows Temporal A, Temporal B, labels, BIT, SNUNet, Changeformer, LGPNet, ConvTransNet, and the experimental results of the proposed DynaNet. Figure 6 demonstrates the qualitative experimental results of DynaNet compared to other state-of-the-art methods on the WHU-CD dataset.

Figure 8 shows that our method significantly reduces the regions of false positives (FP) and false negatives (FN). Specifically, DynaNet effectively avoids the interference of shadows and environmental factors in the detection results. In challenging cases where building colors closely match the surrounding environment, other methods often struggle to detect the disappearance of buildings, leading to missed detections (green areas). In contrast, DynaNet effectively reduces missed detections and accurately locates the change regions. These findings strongly validate the superior performance of DynaNet on the WHU-CD dataset, demonstrating its outstanding ability in complex change scenarios.

Figure 9 shows the visual comparison of DynaNet with other methods on the LEVIR-CD dataset. The LEVIR-CD dataset contains many dense urban building changes, with buildings having irregular shapes. To verify the model’s effectiveness, we selected several images with different object shapes and distributions for comparison. From Figure 9, it can be seen that DynaNet performs better in detecting irregular change targets and can accurately detect small targets at the edges of images. Moreover, our method exhibits strong adaptability in handling simple and complex objects, significantly improving the precision of change detection.

Figure 10 presents the visual comparison results of DynaNet and other methods on the Inner-CD dataset. Unlike datasets such as LEVIR-CD and WHU-CD, the bi-temporal images in Inner-CD contain many buildings, and some buildings undergo spatial position or morphological changes. Such complex change patterns pose a more significant challenge to existing methods. From the visualization results, DynaNet performs exceptionally well in detecting complex building changes and can more accurately capture buildings’ spatial displacement and shape changes, significantly reducing false positives (FP) and false negatives (FN). In comparison, other methods tend to generate misdetections or missed detections in densely built-up areas, particularly lacking in fine-grained structural change detection. Additionally, DynaNet excels in handling edge details, maintaining the integrity of buildings, and avoiding boundary-blurring issues.

### 4.4. Ablation Experiments and Visualiztion

We conducted ablation experiments on three datasets, focusing on validating the contribution of each module in DynaNet. The experiments were performed through module splitting and replacement, aiming to assess the impact of each module on the model’s performance. As shown in Table 4, NO.1 represents the original baseline model. Compared to NO.2, NO.2, which introduces the DFE, significantly improves the model’s performance, with F1-scores and IOU values increasing by 2.24%, 2.89%, 1.40% and 3.84%, 6.97%, 3.74% across the three datasets, respectively. This indicates that the DFE module can effectively capture the change features between bi-temporal images, enhancing the model’s ability to recognize complex change areas. NO.7, based on NO.2, adds the CAM, resulting in F1-score improvements of 4.00% and 9.11% and an increase in IOU values of 6.27% and 10.92%. The CAM module leverages global contextual information to enhance detail recognition and noise suppression, significantly improving the model’s detection accuracy. Further, NO.8, built upon NO.7, introduces the MBAFM, enhancing the change detection performance. The F1-scores increased by 1.70% and 1.69%, and the IOU values rose by 2.09% and 3.06%. The MBAFM module strengthens the fusion of multi-scale features, improving the model’s ability to recognize building change boundaries and reducing false detection occurrences.In summary, each module in DynaNet plays a key role in improving the model’s overall performance, reducing false detections, and minimizing noise interference, further validating the effectiveness of these modules in complex change detection tasks.

Table 5 details the outcomes of our ablation analysis on various configurations of the DEConv and CAM modules within the TVRE framework. The findings indicate that both the DEConv and CAM components enhance the model’s effectiveness through unique mechanisms, and integrating them produces the optimal outcome. Specifically, the DEConv module enhances the model’s ability to extract and refine feature maps, improving the detection of subtle changes. As a result, it leads to an increase in both precision and recall, particularly in the presence of fine-grained changes. On the other hand, CAM focuses on global contextual information, enabling the model to capture broader relationships across the image. This helps suppress background noise and improve the detection of change regions. However, relying solely on CAM may lead to a slight reduction in precision when fine details are more important. When DEConv and CAM are combined, their strengths complement each other. DEConv refines local features, while CAM optimizes global context, increasing model robustness to small and large changes. This combination enhances the model’s overall performance, resulting in higher F1- and IOU scores across all datasets, as it addresses both local feature extraction and global contextual understanding. Thus, the combination of DEConv and CAM achieves the best performance by balancing local detail enhancement with global context integration.

**Figure 11:**
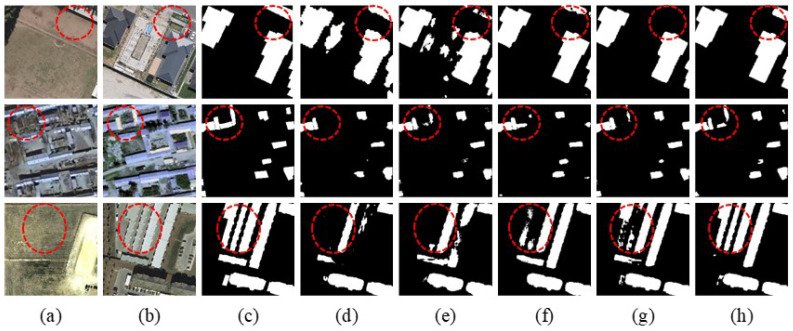
Ablation of Different Modules of DynaNet on Three Datasets. (**a**) Temporal A, (**b**) Temporal B, (**c**) Label, (**d**) Base, (**e**) Base + CAM, (**f**) Base + MBAFM, (**g**) Base + CAM + DFE, (**h**) Base + CAM + DFE + MBAFM.

Figure 11 shows the prediction results of DynaNet with different ablation modules on the LEVIR-CD, WHU-CD and Inner-CD datasets. From top to bottom, the examples for each dataset are presented. (a) and (b) represent remote sensing images before and after the change, (c) show the ground-truth change labels, and (d) display the prediction results of the baseline model. From (e) to (h), key components such as CAM, MBAFM and DFE are progressively introduced. As these modules are added, the detection results of the building change areas become more complete and the impact of false changes is effectively suppressed. This shows that each module plays a positive role in enhancing the change detection performance.

### 4.5. Efficiency Comparison

As indicated by the experiments, our proposed method surpasses the accuracy of the latest building change detection techniques on all three datasets. In this study, we employ the number of model parameters and floating-point operations per second (FLOPs) as metrics for model complexity and computational cost. The number of parameters reflects a model’s ability to learn and represent features, while FLOPs measure the computational effort required for a single forward pass, indicating the model’s efficiency and potential inference speed. Together, these metrics provide a balanced assessment of a model’s representational capability and computational efficiency—particularly crucial for constructing change detection tasks involving high-resolution images and timely processing. Table 6 presents these findings, with all methods standardized to an input size of 256 × 256 × 3 to ensure a consistent evaluation.

**Figure 12:**
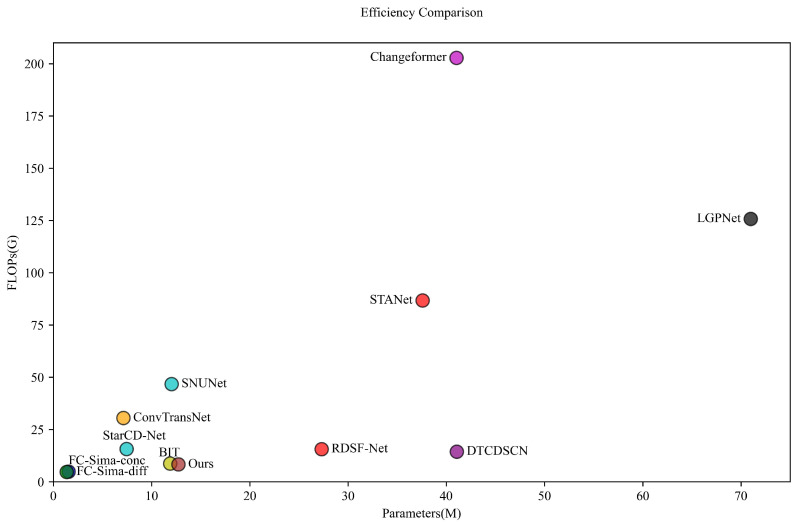
Visualization comparison of the complexity and number of parameters across different models.

The experimental results show that DynaNet improves the F1-score for building change detection accuracy and maintains a relatively low model complexity. This advantage is primarily attributed to the collaborative effect of the DFE, the CAM, and the MBAFM, which enable the model to more precisely extract and fuse multi-scale features, thereby enhancing the ability to recognize building change areas. Although methods such as ConvTransNet and DTCDSCN have slightly lower computational costs than DynaNet, their detection accuracy is relatively lower. Furthermore, DynaNet does not significantly increase the number of trainable parameters or floating-point operations, indicating that it achieves high accuracy while maintaining good computational efficiency. DynaNet enhances building change detection accuracy through efficient feature extraction and fusion strategies without significantly increasing computational complexity. We compare computational complexity and parameter count in Figure 12 to visually demonstrate the model complexity of different methods.

## 5. Discussion

This paper proposes DynaNet, a novel deep neural network approach aimed at identifying changes in buildings from remote sensing data. Extensive experiments have demonstrated that DynaNet performs exceptionally well on a variety of datasets. It not only surpasses traditional CNN-based methods but also outperforms various Transformer models with significantly larger parameter counts, demonstrating promising capabilities for real-world usage and further advancements. The excellent performance of DynaNet can be attributed to three key design features, outlined as follows:

(1) By introducing the DFE module, DynaNet effectively filters out irrelevant features and focuses on detecting meaningful changes. The cross-temporal gating mechanism within DFE allows the model to selectively enhance relevant changes while suppressing background noise, resulting in improved change detection accuracy. This contributes significantly to the model’s robustness in handling complex change scenarios, especially in cases where objects undergo shape or spatial changes.
(2) The CAM module brings global context into feature fusion, greatly enhancing the model’s capacity to concentrate on key areas of change. Global attention helps the network overcome challenges like environmental interference and subtle changes within remote sensing imagery that are often hard to identify. The global context CAM provides ensures that the model effectively captures important change signals, boosting detection precision and recall.
(3) The dual-branch attention fusion mechanism leverages self-attention and cross-attention, allowing the model to model long-range dependencies and interactions between scales and regions. This capability is particularly important for building change detection, as buildings and surrounding structures often interact, and their changes may span various spatial scales. These attention mechanisms enable DynaNet to precisely identify building changes, even in challenging scenarios with complex building layouts and diverse environmental contexts.

Despite these strengths, several limitations and avenues for future work remain:

(1) Performance in complex environments: While DynaNet performs exceptionally well in typical building change detection tasks, its performance may be challenged in highly complex environments, such as areas with dynamic weather conditions, seasonal vegetation changes, or rapid urban development. Future work could explore integrating more advanced pre-trained vision models or multi-modal data to further enhance robustness and accuracy without altering the core structure of DynaNet.
(2) Model complexity and computational cost: DynaNet achieves superior performance by combining modules such as DFE, CAM, and MBAFM. However, this modular design also increases computational complexity and memory requirements, which may limit real-time applications or deployment on resource-constrained devices. Future research could focus on designing more lightweight architectures, pruning redundant connections, or optimizing attention mechanisms to reduce computational cost while maintaining performance.
(3) Generalization across datasets: Although DynaNet demonstrates strong performance on the Inner-CD dataset, its generalization capability across different geographic regions or imaging conditions has not been fully validated. Future studies could investigate cross-dataset evaluation and domain adaptation techniques to ensure broader applicability.

## 6. Conclusions

This paper proposes a new dataset for change detection, Inner-CD, and the DynaNet architecture. The introduction of Inner-CD provides high-quality data for deep learning-based building change detection models, especially for handling more complex and dynamic building transformations. It aims to address the limitations of current datasets, where publicly available datasets typically contain one time point with dense buildings and another with sparse buildings. DynaNet utilizes the DFE module to enhance dynamic feature extraction from bi-temporal images, effectively filtering out irrelevant features and improving change detection accuracy. The CAM combines global contextual information to suppress background noise while amplifying focus on the change areas, significantly improving detection accuracy. The MBAFM module further captures the intrinsic relationships between multi-scale features through self-attention and cross-attention mechanisms, optimizing the segmentation of change areas. Extensive experiments conducted on multiple high-resolution remote sensing datasets show that DynaNet outperforms existing methods, achieving exceptional performance in qualitative and quantitative evaluations. Ablation studies confirm each module’s significant contribution to the model’s overall performance, particularly in capturing fine-grained changes and reducing background noise interference. In conclusion, DynaNet offers a novel and effective solution for the challenge of detecting changes in remote sensing data, making change detection more precise in real-world applications.

However, DynaNet is not without its drawbacks. Future research will focus on incorporating more recent and sophisticated pre-trained models to enhance its capabilities in particularly challenging change scenarios—for instance, those with dynamic environmental factors or rapidly evolving landscapes. Additionally, model optimization techniques such as pruning or distillation will be investigated to reduce computational complexity, making DynaNet suitable for real-time deployment on resource-constrained edge devices while maintaining high detection accuracy.

## Figures and Tables

**Table 1:** Comparison of different change detection methods on the LEVIR-CD dataset, with bold values representing the best experimental results.

Methods	F1 (%)	Pre (%)	Rec (%)	IOU (%)
FC-Sima-conc [19]	86.31	89.53	83.31	77.21
FC-Sima-diff [19]	87.35	89.53	82.45	75.14
STANet [23]	89.17	90.68	87.70	80.45
SNUNet [20]	88.30	91.25	85.55	79.06
Changeformer [29]	89.82	91.85	87.88	81.52
BIT [33]	89.96	91.74	88.25	81.76
StarCD-Net [54]	90.23	91.43	90.15	82.30
LGPNet [35]	89.37	92.13	86.32	80.74
DMATNet [55]	89.97	90.78	89.17	81.83
DTCDSCN [44]	87.67	88.53	86.83	78.05
ConvTransNet [31]	90.43	91.47	87.64	82.56
WNet [32]	90.67	91.16	90.18	82.93
RDSF-Net [56]	91.23	91.34	90.45	83.79
**DynaNet (Ours)**	**92.38**	**93.45**	**91.46**	**84.57**

**Table 2:** Comparison of different change detection methods on the WHU-CD dataset, with bold values representing the best experimental results.

Methods	F1 (%)	Pre (%)	Rec (%)	IOU (%)
FC-Sima-conc [19]	84.76	83.62	86.45	73.56
FC-Sima-diff [19]	85.77	86.53	85.02	75.07
STANet [23]	88.23	89.40	87.10	78.85
SNUNet [20]	89.50	87.60	88.49	79.06
Changeformer [29]	89.47	93.33	83.79	80.52
BIT [33]	89.96	92.24	88.25	83.48
StarCD-Net [54]	92.35	91.43	90.74	85.30
LGPNet [35]	87.07	90.84	85.53	79.74
DMATNet [55]	90.88	91.38	89.68	81.70
DTCDSCN [44]	71.95	85.13	85.12	77.06
ConvTransNet [31]	92.11	92.66	91.57	85.38
WNet [32]	91.25	92.37	90.15	83.91
RDSF-Net [56]	92.14	**94.65**	90.53	85.67
**DynaNet (Ours)**	**94.35**	93.28	**92.46**	**87.57**

**Table 3:** Comparison of different change detection methods on the Inner-CD dataset, with bold values representing the best experimental results.

Methods	F1 (%)	Pre (%)	Rec (%)	IOU (%)
FC-Sima-conc [19]	75.94	73.99	77.99	61.26
FC-Sima-diff [19]	78.79	77.54	78.32	68.07
STANet [23]	83.16	90.04	78.08	71.78
SNUNet [20]	86.36	87.60	85.16	75.25
Changeformer [29]	86.54	89.26	83.98	76.27
BIT [33]	85.15	87.46	84.93	75.68
StarCD-Net [54]	85.41	85.07	86.26	76.53
LGPNet [35]	85.35	84.75	86.48	74.52
DMATNet [55]	87.48	92.24	83.19	77.75
DTCDSCN [44]	85.00	84.87	85.32	73.91
ConvTransNet [31]	86.73	88.52	85.29	76.79
WNet [32]	87.78	89.73	85.92	78.23
RDSF-Net [56]	87.24	89.67	84.75	77.96
**DynaNet (Ours)**	**90.92**	**93.80**	**87.84**	**80.37**

**Table 4:** Ablation study results on the LEVIR-CD, WHU-CD, and Inner-CD Datasets. Bold values represent the best experimental results. ✓ indicates that the module is introduced into the baseline model.

NO.	DFE	CAM	MBAFM	LEVIR-CD		WHU-CD		Inner-CD	
				F1(%)	IOU(%)	F1(%)	IOU(%)	F1(%)	IOU(%)
1				83.29	71.37	77.65	63.58	78.71	65.42
2	✓			85.53	75.21	80.54	70.55	80.11	69.16
3		✓		86.71	75.00	81.94	72.36	81.45	68.42
4			✓	87.01	77.59	84.34	75.91	83.27	70.34
5	✓		✓	89.01	79.59	88.62	83.89	86.79	73.17
6		✓	✓	90.21	81.48	90.94	81.28	87.94	75.36
7	✓	✓		90.72	82.21	92.29	85.69	88.85	78.10
8	✓	✓	✓	**92.38**	**84.57**	**94.35**	**87.57**	**90.92**	**80.37**

**Table 5:** Ablation experimental results of DFE on three datasets, with bold values representing the best experimental results. NO.1 in the table corresponds to NO.6 in Table 4.

NO.	DEConv	CAM	LEVIR-CD		WHU-CD		Inner-CD	
			F1(%)	IOU(%)	F1(%)	IOU(%)	F1(%)	IOU(%)
1			90.21	81.48	90.94	81.28	87.94	75.36
2		✓	91.96	83.56	93.13	86.25	88.73	78.82
3	✓		90.67	82.01	92.36	83.72	88.42	77.54
4	✓	✓	**92.38**	**84.57**	**94.35**	**87.57**	**90.92**	**80.37**

**Table 6:** Experimental comparison of the complexity and number of parameters across different models.

Methods	Params (M)	Flops (G)	ΔF1 (%)
FC-Sima-conc [19]	1.55	4.86	−14.98
FC-Sima-diff [19]	**1.35**	**4.73**	−12.13
STANet [23]	37.59	86.77	−7.76
SNUNet [20]	12.03	46.79	−5.13
Changeformer [29]	41.03	202.86	−4.38
BIT [33]	11.89	8.71	−5.77
LGPNet [35]	70.99	125.79	−3.57
StarCD-Net [54]	7.46	15.74	−5.51
DTCDSCN [44]	41.07	14.42	−5.92
ConvTransNet [31]	7.13	30.53	−3.19
RDSF-Net [56]	27.30	15.60	−3.68
**DynaNet (Ours)**	12.72	8.36	**0**

## Data Availability

The original contributions presented in the study are included in the article, further inquiries can be directed to the corresponding author.
